# Tetra­aqua­bis(biuret-κ^2^ *O*,*O*′)yttrium(III) trichloride

**DOI:** 10.1107/S1600536808008659

**Published:** 2008-04-02

**Authors:** William T. A. Harrison

**Affiliations:** a Department of Chemistry, University of Aberdeen, Meston Walk, Aberdeen AB24 3UE, Scotland

## Abstract

In the title compound, [Y(C_2_H_5_N_3_O_2_)_2_(H_2_O)_4_]Cl_3_, the Y^3+^ ion (site symmetry 2) is bonded to eight O atoms (arising from two *O*,*O*′-bidentate biuret mol­ecules and four water mol­ecules) in a distorted square-anti­prismatic arrangement. A network of N—H⋯O, N—H⋯Cl and O—H⋯Cl hydrogen bonds help to establish the packing, leading to a three-dimensional network. One of the chloride ions has site symmetry 2.

## Related literature

For related structures, see: Carugo *et al.* (1992 ▶); Haddad (1987 ▶, 1988 ▶). For related literature, see: Bernstein *et al.* (1995 ▶). For valence-sum calculations, see: Brese & O’Keeffe (1991 ▶).
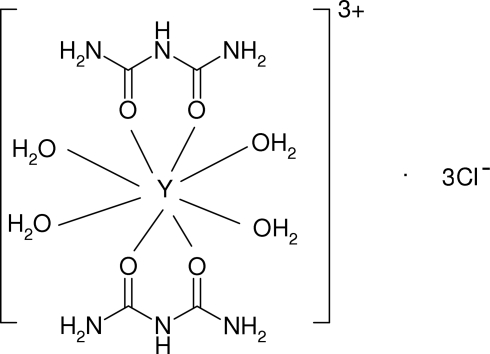


## Experimental

### 

#### Crystal data

[Y(C_2_H_5_N_3_O_2_)_2_(H_2_O)_4_]Cl_3_
*M* *_r_* = 473.50
Monoclinic, 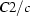

*a* = 7.6510 (4) Å
*b* = 13.2534 (7) Å
*c* = 17.2547 (9) Å
β = 100.817 (1)°
*V* = 1718.57 (16) Å^3^
*Z* = 4
Mo *K*α radiation
μ = 3.90 mm^−1^
*T* = 293 (2) K
0.36 × 0.24 × 0.14 mm

#### Data collection

Bruker SMART1000 CCD diffractometer
Absorption correction: multi-scan (*SADABS*; Bruker, 1999 ▶) *T*_min_ = 0.340, *T*_max_ = 0.611 (expected range = 0.322–0.579)
8075 measured reflections
3105 independent reflections
2464 reflections with *I* > 2σ(*I*)
*R*_int_ = 0.019

#### Refinement

*R*[*F* ^2^ > 2σ(*F* ^2^)] = 0.025
*wR*(*F* ^2^) = 0.059
*S* = 0.98
3105 reflections
101 parameters
H-atom parameters constrained
Δρ_max_ = 0.37 e Å^−3^
Δρ_min_ = −0.34 e Å^−3^

### 

Data collection: *SMART* (Bruker, 1999 ▶); cell refinement: *SAINT* (Bruker, 1999 ▶); data reduction: *SAINT*; program(s) used to solve structure: *SHELXS97* (Sheldrick, 2008 ▶); program(s) used to refine structure: *SHELXL97* (Sheldrick, 2008 ▶); molecular graphics: *ORTEP-3* (Farrugia, 1997 ▶); software used to prepare material for publication: *SHELXL97*.

## Supplementary Material

Crystal structure: contains datablocks I, global. DOI: 10.1107/S1600536808008659/tk2260sup1.cif

Structure factors: contains datablocks I. DOI: 10.1107/S1600536808008659/tk2260Isup2.hkl

Additional supplementary materials:  crystallographic information; 3D view; checkCIF report

## Figures and Tables

**Table 1:** Selected bond lengths (Å)

Y1—O1	2.3157 (11)
Y1—O2	2.3349 (11)
Y1—O4	2.3536 (12)
Y1—O3	2.3660 (10)

**Table 2:** Hydrogen-bond geometry (Å, °)

*D*—H⋯*A*	*D*—H	H⋯*A*	*D*⋯*A*	*D*—H⋯*A*
N1—H1⋯O1^i^	0.86	2.10	2.9111 (18)	157
N1—H2⋯Cl1^ii^	0.86	2.39	3.1897 (17)	155
N2—H3⋯Cl1^ii^	0.86	2.53	3.3184 (14)	153
N3—H4⋯Cl1^iii^	0.86	2.54	3.3633 (15)	161
N3—H5⋯Cl1^iv^	0.86	2.54	3.3232 (15)	151
O3—H6⋯Cl1^v^	0.80	2.39	3.1607 (12)	161
O3—H7⋯Cl2^vi^	0.79	2.27	3.0504 (12)	168
O4—H8⋯Cl1	0.75	2.45	3.2028 (13)	177
O4—H9⋯Cl2	0.80	2.40	3.1238 (12)	150

Symmetry codes: (i) 
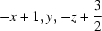
; (ii) 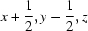; (iii) 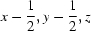; (iv) 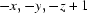; (v) 
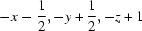
; (vi) 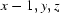.

**Fig. 1.**
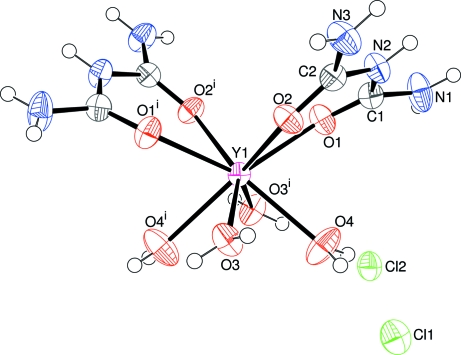
View of the molecular structure of (I) showing 50% displacement ellipsoids (arbitrary spheres for the H atoms). Symmetry code: (i) -x, y, 3/2 - z.

**Fig. 2.**
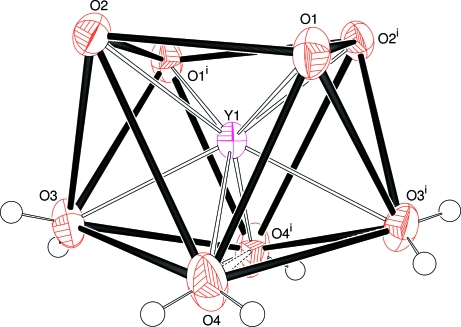
Detail of (I) showing the distorted square antiprismatic coordination of the Y atom. Symmetry code: (i) -x, y, 3/2 - z.

**Fig. 3.**
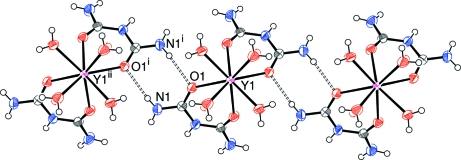
Fragment of a [100] chain of cations in (I) with the hydrogen bonds indicated by double-dashed lines. Symmetry codes: (i) 1 - x, y, 3/2 - z; (ii) x + 1, y, z.

## supplementary crystallographic information

### Comment

No complexes of yttrium(III) with biuret (biur), H_2_N—CO—NH—CO—NH_2_ (or C_2_H_5_N_3_O_2_) have been structurally characterized. The structures of two samarium-biuret complexes, Sm(biur)_4_.(NO_3_)_3_ (Haddad, 1987) and Sm(biur)_4_.(ClO_4_)_3_ (Haddad, 1988) have been described. In both cases, an SmO_8_ square antiprismatic coordination arises for the metal ion. Based on X-ray photographs, it was suggested that all the Ln(biur)_4_.(NO_3_)_3_ and Ln(biur)_4_.(ClO_4_)_3_ compounds are isostructural with their samarium prototypes. In this paper, we describe the synthesis and structure of the title compound, (I).

Compound (I) is an ionic salt containing a new [Y(biur)_2_(H_2_O)_4_]^3+^ complex ion. The complete complex ion is generated by crystallographic 2-fold symmetry, with the Y atom lying on the rotation axis. Two uncoordinated chloride ions, one of which has crystallographic site symmetry 2, complete the structure of (I), Fig. 1.

The resulting YO_8_ polyhedral geometry in (I) (Table 1) is a distorted square antiprism (Fig. 2). The nominal square face formed by atoms O1, O2, O1^i^ and O2^i^ (i = -*x*, *y*, 3/2 - *z*) is reasonably regular, but the second face formed by the four water molecules (O3, O4, O3^i^ and O4^i^) is much more distorted, and the diagonal O3···O3^i^ and O4···O4^i^ distances of 4.223 (2)Å and 3.5197 (19) Å, respectively, are very different. The Y1 atom deviates from the mean planes of O1/O2/O1^i^/O2^i^ and O3/O4/O3^i^/O4^i^ by 1.1616 (8) Å and 1.3151 (9) Å, respectively. The two O atom mean planes are constrained to be parallel by symmetry. The bond valence sum (Brese & O'Keeffe, 1991) for Y1 of 3.34 in (I) indicates that its valence requirement is easily satisfied by this geometry.

The O,*O*-bidenate coordination of the biuret molecule to the yttrium ion in (I) results in a six-membered chelate ring that is non-planar. As noted previously (Carugo *et al.*, 1992), the biuret molecule can be regarded as two planar amide fragments linked by the NH bridge. Here, the dihedral angle betwen the N1/C1/O1/N2 and N2/C2/O2/N3 units is 5.06 (10)°. The yttrium cation deviates from the N1/C1/O1/N2 and N2/C2/O2/N3 mean planes by 0.894 (4) Å and 0.606 (4) Å, respectively.

The component species in (I) are linked by a dense array of N—H···O, N—H···Cl and O—H···Cl hydrogen bonds (Table 2) resulting in a three-dimensional network. Of note is the N—H···O hydrogen bond, which results in [100] chains (Fig. 3) of cations, in which *R*^2^_2_(8) loops (Bernstein *et al.*, 1995) linking the molecules are apparent.

### Experimental

0.1 *M* Aqueous solutions of YCl_3_ (10 ml) and biuret (10 ml) were mixed and a small quantity of dilute hydrochloric acid was added, to result in a colourless solution. Colourless blocks of (I) grew over several days as the water slowly evaporated.

### Refinement

The N-bound hydrogen atoms were geometrically placed (N—H = 0.88 Å) and refined as riding with *U*_iso_(H) = 1.2*U*_eq_(N). The water H atoms were located in difference maps and refined as riding in their as-found relative positions with *U*_iso_(H) = 1.2*U*_eq_(O); see Table 2 for O-H distances.

### Crystal data

**Table:**

[Y(C_2_H_5_N_3_O_2_)_2_(H_2_O)_4_]Cl_3_	*F*_000_ = 952
*M**_r_* = 473.50	*D*_x_ = 1.830 Mg m^−^^3^
Monoclinic, *C*2/*c*	Mo *K*α radiation λ = 0.71073 Å
Hall symbol: -C 2yc	Cell parameters from 3637 reflections
*a* = 7.6510 (4) Å	θ = 3.1–32.4º
*b* = 13.2534 (7) Å	µ = 3.90 mm^−^^1^
*c* = 17.2547 (9) Å	*T* = 293 (2) K
β = 100.817 (1)º	Block, colourless
*V* = 1718.57 (16) Å^3^	0.36 × 0.24 × 0.14 mm
*Z* = 4	

### Data collection

**Table:**

Bruker SMART1000 CCD diffractometer	3105 independent reflections
Radiation source: fine-focus sealed tube	2464 reflections with *I* > 2σ(*I*)
Monochromator: graphite	*R*_int_ = 0.019
*T* = 293(2) K	θ_max_ = 32.6º
ω scans	θ_min_ = 2.4º
Absorption correction: multi-scan(SADABS; Bruker, 1999)	*h* = −11→11
*T*_min_ = 0.340, *T*_max_ = 0.611	*k* = −20→19
8075 measured reflections	*l* = −16→26

### Refinement

**Table:**

Refinement on *F*^2^	Secondary atom site location: difference Fourier map
Least-squares matrix: full	Hydrogen site location: difmap (O-H) and geom (N-H)
*R*[*F*^2^ > 2σ(*F*^2^)] = 0.025	H-atom parameters constrained
*wR*(*F*^2^) = 0.059	*w* = 1/[σ^2^(*F*_o_^2^) + (0.0303*P*)^2^] where *P* = (*F*_o_^2^ + 2*F*_c_^2^)/3
*S* = 0.98	(Δ/σ)_max_ < 0.001
3105 reflections	Δρ_max_ = 0.37 e Å^−^^3^
101 parameters	Δρ_min_ = −0.34 e Å^−^^3^
Primary atom site location: structure-invariant direct methods	Extinction correction: none

### Special details

**Table:**

Geometry. All e.s.d.'s (except the e.s.d. in the dihedral angle between two l.s. planes) are estimated using the full covariance matrix. The cell e.s.d.'s are taken into account individually in the estimation of e.s.d.'s in distances, angles and torsion angles; correlations between e.s.d.'s in cell parameters are only used when they are defined by crystal symmetry. An approximate (isotropic) treatment of cell e.s.d.'s is used for estimating e.s.d.'s involving l.s. planes.
Refinement. Refinement of *F*^2^ against ALL reflections. The weighted *R*-factor *wR* and goodness of fit *S* are based on *F*^2^, conventional *R*-factors *R* are based on *F*, with *F* set to zero for negative *F*^2^. The threshold expression of *F*^2^ > σ(*F*^2^) is used only for calculating *R*-factors(gt) *etc*. and is not relevant to the choice of reflections for refinement. *R*-factors based on *F*^2^ are statistically about twice as large as those based on *F*, and *R*- factors based on ALL data will be even larger.

### Fractional atomic coordinates and isotropic or equivalent isotropic displacement parameters (Å^2^)

**Table:**

	*x*	*y*	*z*	*U*_iso_*/*U*_eq_	
Y1	0.0000	0.107273 (15)	0.7500	0.02386 (6)	
Cl1	−0.04205 (5)	0.33346 (3)	0.50811 (2)	0.03905 (10)	
Cl2	0.5000	0.29705 (5)	0.7500	0.03992 (14)	
O1	0.25038 (14)	0.02884 (9)	0.72093 (7)	0.0348 (3)	
O2	−0.08317 (13)	0.01042 (8)	0.63600 (7)	0.0329 (2)	
N1	0.45654 (18)	−0.01014 (13)	0.64850 (10)	0.0476 (4)	
H1	0.5399	0.0182	0.6817	0.057*	
H2	0.4804	−0.0382	0.6068	0.057*	
N2	0.17105 (17)	−0.05973 (11)	0.60525 (8)	0.0332 (3)	
H3	0.2113	−0.0980	0.5724	0.040*	
N3	−0.10477 (19)	−0.11253 (11)	0.54451 (9)	0.0410 (3)	
H4	−0.2191	−0.1099	0.5351	0.049*	
H5	−0.0506	−0.1546	0.5193	0.049*	
C1	0.2922 (2)	−0.01064 (12)	0.66153 (10)	0.0308 (3)	
C2	−0.0118 (2)	−0.05156 (11)	0.59806 (9)	0.0295 (3)	
O3	−0.23875 (15)	0.18780 (9)	0.66560 (7)	0.0377 (3)	
H6	−0.2731	0.1747	0.6201	0.045*	
H7	−0.3170	0.2159	0.6812	0.045*	
O4	0.11867 (16)	0.22520 (10)	0.67195 (8)	0.0519 (4)	
H8	0.0828	0.2492	0.6326	0.062*	
H9	0.2176	0.2465	0.6742	0.062*	

### Atomic displacement parameters (Å^2^)

**Table:**

	*U*^11^	*U*^22^	*U*^33^	*U*^12^	*U*^13^	*U*^23^
Y1	0.02161 (9)	0.02849 (10)	0.02041 (9)	0.000	0.00117 (7)	0.000
Cl1	0.0364 (2)	0.0491 (2)	0.0297 (2)	0.00110 (17)	0.00118 (17)	−0.00610 (18)
Cl2	0.0316 (3)	0.0479 (3)	0.0418 (3)	0.000	0.0107 (2)	0.000
O1	0.0255 (5)	0.0467 (6)	0.0309 (6)	0.0049 (5)	0.0014 (5)	−0.0080 (5)
O2	0.0282 (5)	0.0358 (6)	0.0327 (6)	0.0034 (5)	0.0004 (5)	−0.0092 (5)
N1	0.0266 (7)	0.0778 (11)	0.0394 (8)	−0.0078 (7)	0.0089 (6)	−0.0204 (8)
N2	0.0266 (6)	0.0415 (7)	0.0315 (7)	0.0016 (6)	0.0053 (5)	−0.0088 (6)
N3	0.0321 (7)	0.0459 (8)	0.0426 (8)	−0.0014 (6)	0.0012 (6)	−0.0179 (7)
C1	0.0271 (7)	0.0350 (8)	0.0293 (8)	0.0013 (6)	0.0025 (6)	0.0004 (7)
C2	0.0290 (7)	0.0320 (7)	0.0261 (7)	−0.0009 (6)	0.0021 (6)	0.0012 (6)
O3	0.0353 (6)	0.0498 (7)	0.0253 (6)	0.0145 (5)	−0.0011 (5)	−0.0026 (5)
O4	0.0390 (7)	0.0677 (9)	0.0436 (7)	−0.0157 (6)	−0.0061 (6)	0.0281 (7)

### Geometric parameters (Å, °)

**Table:**

Y1—O1^i^	2.3157 (11)	N1—H2	0.8600
Y1—O1	2.3157 (11)	N2—C1	1.374 (2)
Y1—O2	2.3349 (11)	N2—C2	1.385 (2)
Y1—O2^i^	2.3349 (11)	N2—H3	0.8600
Y1—O4^i^	2.3536 (12)	N3—C2	1.329 (2)
Y1—O4	2.3536 (12)	N3—H4	0.8600
Y1—O3^i^	2.3660 (10)	N3—H5	0.8600
Y1—O3	2.3660 (10)	O3—H6	0.7990
O1—C1	1.2450 (19)	O3—H7	0.7934
O2—C2	1.2392 (18)	O4—H8	0.7541
N1—C1	1.318 (2)	O4—H9	0.8023
N1—H1	0.8600		
			
O1^i^—Y1—O1	126.66 (6)	O4—Y1—O3	71.61 (4)
O1^i^—Y1—O2	80.21 (4)	O3^i^—Y1—O3	126.37 (6)
O1—Y1—O2	71.12 (4)	C1—O1—Y1	135.89 (10)
O1^i^—Y1—O2^i^	71.12 (4)	C2—O2—Y1	137.05 (10)
O1—Y1—O2^i^	80.21 (4)	C1—N1—H1	120.0
O2—Y1—O2^i^	113.30 (6)	C1—N1—H2	120.0
O1^i^—Y1—O4^i^	75.55 (5)	H1—N1—H2	120.0
O1—Y1—O4^i^	147.74 (4)	C1—N2—C2	124.22 (14)
O2—Y1—O4^i^	140.68 (4)	C1—N2—H3	117.9
O2^i^—Y1—O4^i^	87.51 (4)	C2—N2—H3	117.9
O1^i^—Y1—O4	147.74 (4)	C2—N3—H4	120.0
O1—Y1—O4	75.55 (5)	C2—N3—H5	120.0
O2—Y1—O4	87.51 (4)	H4—N3—H5	120.0
O2^i^—Y1—O4	140.68 (4)	O1—C1—N1	122.63 (15)
O4^i^—Y1—O4	96.78 (8)	O1—C1—N2	122.49 (14)
O1^i^—Y1—O3^i^	130.07 (4)	N1—C1—N2	114.87 (15)
O1—Y1—O3^i^	76.19 (4)	O2—C2—N3	122.60 (14)
O2—Y1—O3^i^	145.42 (4)	O2—C2—N2	122.84 (14)
O2^i^—Y1—O3^i^	70.89 (4)	N3—C2—N2	114.49 (15)
O4^i^—Y1—O3^i^	71.61 (4)	Y1—O3—H6	125.5
O4—Y1—O3^i^	73.53 (4)	Y1—O3—H7	123.2
O1^i^—Y1—O3	76.19 (4)	H6—O3—H7	107.8
O1—Y1—O3	130.07 (4)	Y1—O4—H8	133.0
O2—Y1—O3	70.89 (4)	Y1—O4—H9	132.0
O2^i^—Y1—O3	145.42 (4)	H8—O4—H9	94.3
O4^i^—Y1—O3	73.53 (4)		

Symmetry codes: (i) −*x*, *y*, −*z*+3/2.

### Hydrogen-bond geometry (Å, °)

**Table:**

*D*—H···*A*	*D*—H	H···*A*	*D*···*A*	*D*—H···*A*
N1—H1···O1^ii^	0.86	2.10	2.9111 (18)	157
N1—H2···Cl1^iii^	0.86	2.39	3.1897 (17)	155
N2—H3···Cl1^iii^	0.86	2.53	3.3184 (14)	153
N3—H4···Cl1^iv^	0.86	2.54	3.3633 (15)	161
N3—H5···Cl1^v^	0.86	2.54	3.3232 (15)	151
O3—H6···Cl1^vi^	0.80	2.39	3.1607 (12)	161
O3—H7···Cl2^vii^	0.79	2.27	3.0504 (12)	168
O4—H8···Cl1	0.75	2.45	3.2028 (13)	177
O4—H9···Cl2	0.80	2.40	3.1238 (12)	150

Symmetry codes: (ii) −*x*+1, *y*, −*z*+3/2; (iii) *x*+1/2, *y*−1/2, *z*; (iv) *x*−1/2, *y*−1/2, *z*; (v) −*x*, −*y*, −*z*+1; (vi) −*x*−1/2, −*y*+1/2, −*z*+1; (vii) *x*−1, *y*, *z*.
